# Novel mechanism for mesenchymal stem cells in attenuating peritoneal adhesion: accumulating in the lung and secreting tumor necrosis factor α-stimulating gene-6

**DOI:** 10.1186/scrt142

**Published:** 2012-12-06

**Authors:** Nan Wang, Yeqing Shao, Yan Mei, Li Zhang, Qinggang Li, Diangeng Li, Suozhu Shi, Quan Hong, Hongli Lin, Xiangmei Chen

**Affiliations:** 1 State Key Laboratory of Kidney Diseases, Department of Nephrology, Chinese PLA General Hospital and Medical School of Chinese PLA, Beijing 100853, China; 2 Medical College, NanKai University, Tianjin 300071, China; 3 Department of Nephrology, First Affiliated Hospital of Henan University of Science and Technology, Henan 471003, China; 4 Department of Nephrology, the First Affiliated Hospital of Dalian Medical University, Liaoning 116011, China

## Abstract

**Introduction:**

We previously found that mesenchymal stem cells (MSCs) injected intravenously could attenuate peritoneal adhesion by secreting tumor necrosis alpha-stimulating gene (TSG)-6, while MSCs injected intraperitoneally could not. However, the underlying mechanism remains unclear. This study was designed to investigate the means by which MSCs exert their effects.

**Methods:**

Rat bone marrow-derived MSCs/red fluorescent protein (RFP) were injected either intraperitoneally or intravenously into Sprague-Dawley (SD) rats at different time points after peritoneal scraping. Peritoneal adhesions were evaluated macroscopically at day 14 after scraping. The distribution of MSCs injected intraperitoneally or intravenously was traced by two-photon fluorescence confocal imaging and immunofluorescence microscopy. The co-localization of MSCs and macrophages in the lung and the spleen, and the expression of TSG-6 in MSCs trapped in the lung or the spleen were evaluated by immunofluorescence microscopy. The concentration of TSG-6 in serum was evaluated by ELISA. After intravenous injection of TSG-6- small interfering (si) RNA-MSCs, the expression of TSG-6 in MSCs and the concentration of TSG-6 in serum were reevaluated, and peritoneal adhesions were evaluated macroscopically and histologically.

**Results:**

MSCs injected intraperitoneally failed to reduce peritoneal adhesion, and MSCs injected intravenously markedly improved peritoneal adhesion. Two-photon fluorescence confocal imaging showed that MSCs injected intravenously accumulated mainly in the lung, where they remained for seven days, and immunofluorescence microscopy showed few MSCs phagocytosed by macrophages. In contrast, large numbers of MSCs accumulated in the spleen with obvious phagocytosis by macrophages even at 4 hours after intraperitoneal injection. Immunofluorescence microscopy showed that MSCs that accumulated in the lung after intravenous injection could express TSG-6 within 12 hours, but TSG-6-siRNA-MSCs or MSCs accumulated in the spleen after intraperitoneal injection did not. ELISA showed that the concentration of TSG-6 in serum was increased at 4 hours after intravenous injection of MSCs, while there was no increase after injection of TSG-6-siRNA-MSCs or after intraperitoneal injection of MSCs. Moreover, intravenous injection of TSG-6-siRNA-MSCs failed to attenuate peritoneal adhesion.

**Conclusions:**

Our findings suggest that intravenously injected MSCs accumulated in the lung and attenuated peritoneal adhesion by secreting TSG-6, but intraperitoneally injected MSCs were phagocytosed by macrophages in the spleen and failed to attenuate peritoneal adhesion.

## Introduction

Studies demonstrate that mesenchymal stem cells (MSCs) can repair injuries [1,2] and decrease fibrosis in the heart [3], lung [4] and kidney [5]. However, the mechanisms remain controversial. Researchers believe that the effect is mediated by an increase in mitogenic [6], anti-inflammatory, anti-apoptotic, immunosuppressive and anti-fibrogenic factors [2,7], as well as differentiation into specific cells [8].

Peritoneal fibrosis and adhesion are the major causes of ultrafiltration failure in peritoneal dialysis (PD) patients [9], and postoperative peritoneal adhesions [10] are also problematic. We previously found that MSCs injected intravenously attenuated peritoneal adhesion by repairing mesothelial cells, as well as reducing inflammation and fibrosis. Rather than the engraftment, the secretion of multifunctional anti-inflammatory TNFα-stimulating gene (TSG)-6 by MSCs plays a major role in this effect [11] but MSCs injected intraperitoneally failed to attenuate peritoneal adhesion. Studies have shown that MSCs injected intraperitoneally are activated by the inflammatory microenvironment of the peritoneal cavity to secrete TSG-6 and attenuate peritonitis induced by zymosan in mice [12]. Another study found that both intraperitoneal and intravenous injection of MSCs suppress corneal inflammation in rats by secreting TSG-6 [13]. Our findings were inconsistent with these studies. We do not have direct evidence that MSCs injected intravenously can secrete TSG-6 and exert effects on the injured peritoneum.

The aim of this research was to investigate the way in which MSCs exert their effects on peritoneal adhesion and to specify the causes for the failure of MSCs injected intraperitoneally. We demonstrated that intravenously injected MSCs accumulated in the lung and attenuated peritoneal adhesion by secreting TSG-6 into the blood, while intraperitoneally injected MSCs were phagocytosed by splenic macrophages.

## Methods

### Acute peritoneal adhesion rat models

This study was approved by the Ethics Committee of The General Hospital of the People's Liberation Army (Permit Number: 2010-X-3-28) with animal care performed strictly according to established institutional guidelines. All surgery was performed under pentobarbital anesthesia. Scrape-induced peritoneal adhesions were created in healthy male Sprague-Dawley (SD) rats weighing 200 g to 250 g. All animals were obtained from the Experimental Animal Center of the Academy of Military Medical Sciences (Beijing, China) and housed at constant room temperature with a 12-hour light/dark cycle. Standard rodent chow and water were provided *ad libitum*. The animals were acclimated for seven days before initiating the experiment.

Surgical procedures were conducted by a single surgeon under aseptic conditions in the Laboratory Animal Unit. Rats were anesthetized with a 2% pentobarbital (30 mg/kg) intraperitoneal injection. Briefly, a 2-cm vertical midline incision was made into the abdominal wall and peritoneum. The dorsal and ventral surfaces of the cecum were scraped with dry gauze 20 times over an area of 2 × 2 cm^2 ^until petechial bleeding occurred, and the cecum was then replaced. The parietal peritoneum lateral to the midline incision was scraped 20 times until petechial bleeding occurred. The incision was closed in two layers with 4/0 silk sutures [14]. After surgery, the rats were kept in a single cage and fed a normal diet.

### MSCs culture

SD rat bone marrow-derived MSCs/red fluorescent protein (RFP) was obtained commercially (Cyagen Biosciences, Sunnyvale, CA, USA). The culture was initiated following the manufacturer's instructions. MSCs were placed into 25 cm^2 ^culture flasks and cultured with MSCs growth medium (Cyagen Biosciences, Sunnyvale, CA, USA) at 37°C under 5% CO_2 _and 90% humidity. The medium was changed every three days. Sixth to eighth passage MSCs were used for the experiments. Following previous methods [15], fluorescence-activated cell sorting (FACS) analysis (Beckman Coulter, Indianapolis, IN, USA) was used to examine the representative markers of MSCs (CD45, CD90 (BD Biosciences, San Diego, California, USA); CD11a, CD54 (AbD Serotec, Oxford, UK)), and multilineage differentiation of MSCs was examined under adipogenic and osteogenic differentiation conditions.

### Transfection of MSCs with TSG-6 small interfering RNA (siRNA)

Fifty-percent confluent MSCs were transfected with 20 nM TSG-6-small interfering (si) RNA or the siRNA-negative control (NC) (GenePharma, Shanghai, China) using INTERFERin™(Polyplus-transfection SA, Bioparc, France). At 24 hours after transfection, MSCs were fed with serum-free medium for 24 hours, prior to experiments. To confirm the knockdown of TSG-6, RNA was assayed for TSG-6 by RT-PCR (TSG-6 forward primer: AGTGATGCGTCCGTCACAGCC, reverse primer: AGATGGCTAAACCGTCCAGCTAAGA, product length = 134 bp; GAPDH forward primer: GGCATGGACTGTGGTCATGAG, reverse primer: TGCACCACCAACTGCTTAGC, product length = 87 bp (SBS Genetech, Beijing, China)), and the protein was assayed for TSG-6 by Western blot (primary antibodies TSG-6 (1:50) (Santa Cruz Biotechnology, Santa Cruz, CA, USA)).

### Injection of MSCs or recombinant mouse (rm) TSG-6

At 0, 4, 12, 24, or 48 hours after peritoneal scraping, MSCs (5 × 10^6^) in 1-ml serum-free medium were injected via the tail vein or peritoneum. At 24 hours after peritoneal scraping, TSG-6-siRNA-MSCs, TSG-6-siRNA-NC-MSCs or 3 ng/ml rmTSG-6 (97% homology with rat [16]) (R&D Systems Inc., Minneapolis, MN, USA) in 1-ml serum-free medium (adapted from our previous experiment [11]) were injected via the tail vein. Rats injected with the serum-free medium were the negative control, and the rats without peritoneal scraping were the blank control.

### Two-photon fluorescence confocal imaging of MSCs after injection

A Leica two-photon fluorescence confocal imaging TCS SP5 system (Leica Microsystems, Mannheim, Germany) was used to evaluate the distribution of MSCs after injection. The excitation and emission filter set for green (autofluorescence) detection was 488 nm and 504 to 569 nm, respectively. The excitation and emission filter set for red detection was 543 nm and 555 to 624 nm, respectively. MSCs were injected into rats intraperitoneally or intravenously at 24 hours after scraping. Rats were sacrificed at 4, 12, 24, 48, and 72 hours and 5, 7 days thereafter (*n *= 3 in each group at each time point). Fresh thick tissues of the right lung, right lower liver, spleen, and scraped peritoneum were sampled.

### Immunofluorescence staining of lung and spleen

MSCs, TSG-6-siRNA-MSCs, or TSG-6-siRNA-NC-MSCs were injected into rats intraperitoneally or intravenously at 24 hours after scraping. Rats were sacrificed at 4, 12, 24, 48 and 72 hours and 5, 7 days thereafter (*n *= 3 in each group at each time point). The right lung and the spleen were sampled. Specimens were embedded in optimum cutting temperature (OCT) compound and stored at -80°C until use. Frozen tissues were sectioned every 4 μm and placed on poly-L-lysine precoated slides. The slides were fixed with 4% paraformaldehyde for 5 minutes at room temperature, and for 10 minutes at 4°C. The slides were then blocked with 1% BSA for 30 minutes at room temperature. The following primary antibodies were incubated overnight at 4°C: ED-1 (1:50) (Santa Cruz Biotechnology) and TSG-6 (1:50) (Santa Cruz Biotechnology, sc-30140). Secondary antibodies conjugated with fluorescein isothiocyanate (FITC) (Jackson ImmunoResearch Laboratories, West Grove, PA, USA) were applied for 1 hour at room temperature in a darkened humidified chamber. Finally, the preparations were mounted in fluorescent mounting medium with 4',6-diamidino-2-phenylindole (DAPI). Negative controls did not receive the first antibody. Each tissue section was observed under a confocal laser scanning microscope (Olympus FluoView 1000, Tokyo, Japan) at magnifications of × 600 and × 1800. Three-dimensional imaging was applied, if necessary.

### Enzyme-linked immunosorbent assay (ELISA) of TSG-6 in rat serum

Rats were sacrificed at 4, 12, 24, 48, or 72 hours, or 5, 7, 14 days after MSCs, TSG-6-siRNA-MSCs, or TSG-6-siRNA-NC-MSCs injection intravenously at 24 hours after scraping (*n *= 3 in each group at each time point). Quantification of TSG-6 in serum was performed by ELISA according to the conventional procedure. Absorbance was measured at 450 nm using a microplate reader (Thermo Fisher Scientific, Waltham, MA, USA). TSG-6 concentrations were determined with a standard curve constructed by titrating rmTSG-6. All samples were placed in three replicate wells. A laboratory-made ELISA kit was prepared mainly as follows: a 96-well microplate (Corning, Lowell, MA, USA) was coated with mouse monoclonal antibody to TSG-6 (Santa Cruz Biotechnology, sc-377277), enzyme-labeled secondary antibody was purchased from Dako (Glostrup, Denmark). The correlation coefficiency was 0.995 for the standard curves.

### Macroscopic evaluation of peritoneal adhesions

At 0, 4, 12, 24, or 48 hours after peritoneal scraping, MSCs were injected intraperitoneally. At 24 hours after scraping, MSCs or rmTSG-6 were injected intravenously. Rats were sacrificed on day 14 after scraping (*n *= 6 in each group at each time point). The size and severity of peritoneal adhesions were evaluated macroscopically by an independent observer on a scale of 0 to 4 (0, 0%; 1, <25%; 2, 25% to 49%; 3, 50% to 74%; and 4, 75% to 100% adhesions) using a previously reported scoring system [17].

### Histological analysis of peritoneal adhesions

Rats were sacrificed on day 14 after scraping (*n *= 6 in each group at each time point). The entire fibrous band was sampled. Specimens were fixed in 10% formaldehyde for 24 hours. After dehydration, they were embedded in paraffin, and 3-μm thick cross-sections were stained with Masson's trichrome. Each tissue section was examined by light microscopy (Olympus IX71, Tokyo, Japan) at magnifications of × 100 and × 400. Five randomly selected fields in each section were evaluated by an independent pathologist (at a magnification of × 100). The extent of fibrosis was scored as 0 (negative), 1 (weak), 2 (medium), or 3 (intensive).

### Statistical analysis

Analysis was done using SPSS Statistics, version 17.0.2. Results are presented as mean values ± standard deviations. Multiple comparisons of parametric data were performed using one-way analysis of variance (ANOVA). Nonparametric data were compared with the Mann-Whitney *U*-test to identify differences between groups. A value of *P *<0.05 was considered to indicate statistical significance.

## Results and discussion

### MSCs injected intraperitoneally failed to attenuate peritoneal adhesion

**Figure 1:**
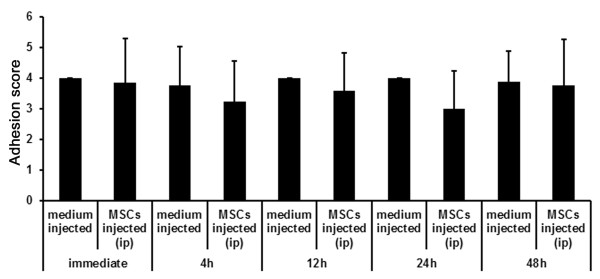
**Effects of intraperitoneally injected mesenchymal stem cells (MSCs) on the severity of acute peritoneal adhesions**. MSCs injected intraperitoneally into rats either immediately or 4, 12, 24, or 48 hours after peritoneal scraping yielded no apparent reduction in adhesions (*n *= 6).

We previously found that MSCs injected intraperitoneally 24 hours after scraping failed to attenuate peritoneal adhesion [11]. One related study reported that MSCs injected intraperitoneally into mice 15 minutes after zymosan administration attenuated peritonitis [12]. Another study reported that both intraperitoneal and intravenous injection of MSCs into rats immediately following injury can suppress corneal inflammation [13]. Our findings were inconsistent with these reports, which injected MSCs as early as possible. We previously found that infiltration of neutrophils (the active inflammation phase) peaked 12 to 24 hours after scraping [11], so we selected an earlier and wider range of time points for injection. At 0, 4, 12, 24, or 48 hours after scraping, MSCs (identifications are shown in Additional file 1, Figure S1) were injected intraperitoneally. On day 14 after scraping, the medium-injected rats demonstrated dense adhesions with high adhesion scores. The rats that had MSCs injected intraperitoneally showed no apparent reduction in adhesions. We observed no significant difference among the groups (Figure 1) (*P *>0.05, *n *= 6, respectively). Therefore, we concluded that MSCs injected intraperitoneally did not attenuate peritoneal adhesion, regardless of the time of injection.

It has become apparent that MSCs repair injured tissues without engraftment [18]. In fact, MSCs secrete a number of cytokines and growth factors that alter the tissue microenvironment. In order to evaluate why MSCs injected intraperitoneally failed to function efficiently, we next investigated the differences in the distribution of intraperitoneally and intravenously injected MSCs.

### MSCs injected intraperitoneally were phagocytosed by macrophages

**Figure 2:**
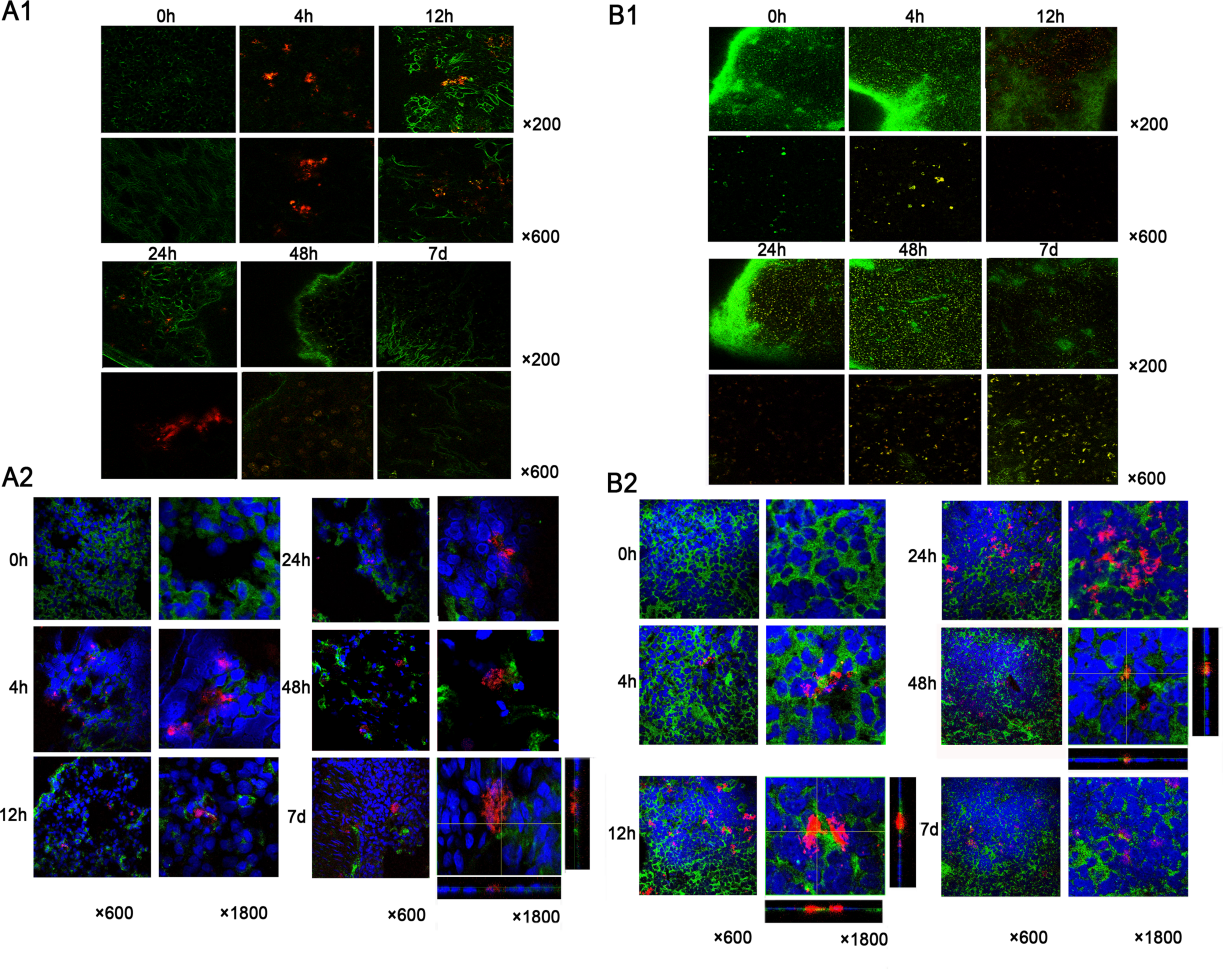
**The distributions of mesenchymal stem cells (MSCs) injected into rats intravenously or intraperitoneally**. **A**. The distribution of MSCs injected intravenously. **A1**. The accumulation of MSCs in the lung tracked by two-photon fluorescence confocal imaging. Red symbolizes red fluorescent protein (RFP); Green symbolizes autofluorescence of the lung. Magnification: × 200, × 600. **A2**. MSCs did not co-localize with macrophages in the lung, as observed by immunofluorescence microscopy. Red symbolizes RFP; Green symbolizes ED-1 of macrophages. Magnification: × 600, × 1800. **B**. The distribution of MSCs injected intraperitoneally. **B1**. The accumulation of MSCs in the spleen tracked by two-photon fluorescence confocal imaging. Red symbolizes RFP; Green symbolizes autofluorescence of the spleen. Magnification: × 200, × 600. **B2**. The majority of MSCs co-localized with macrophages in the spleen, as observed by immunofluorescence microscopy. Red symbolizes RFP; Green symbolizes ED-1 of macrophages. Magnification: × 600, × 1800.

Two-photon microscopy has advantages over traditional confocal fluorescence microscopy, including decreased out of focus photodamage and increased intrinsic optical sectioning and imaging depths [19]. To track the distribution of MSCs after injection intravenously or intraperitoneally, we used two-photon fluorescence confocal imaging to observe the fresh thick tissues of lung, spleen, liver and peritoneum. The findings showed that MSCs injected intravenously accumulated mainly in the interstitial areas of the lung appearing as 'emboli' 4 hours after injection, then decreased gradually and began to accumulate in the spleen and liver, but the RFP signal persisted in the lung for 7 days (Figure 2A1); no signal was found in the injured peritoneum throughout the entire period (data not shown). Immunofluorescence microscopy for RFP and ED-1 double-stained cells showed that few MSCs were phagocytosed by macrophages in the interstitial areas between alveoli of the lung (Figure 2A2). While MSCs injected intraperitoneally accumulated mainly in the spleen appearing as 'debris' 4 hours after injection, the signal increased and peaked at 24 hours after injection (Figure 2B1). The changes of signal in the liver were similar. No signal was found in the lung or the injured peritoneum (data not shown). Immunofluorescence microscopy showed apparent phagocytosis of MSCs by macrophages even at 4 hours after injection (Figure 2B2, Additional file 2, Video S1 and Additional file 3, Video S2).

Our findings were similar to our previous results when we used an *in vivo *imaging system to track the distribution of MSCs [11]. The vast majority of MSCs injected intravenously accumulated in the lungs [18,20]. This distribution may be due to the size of MSCs (20 μm to 30 μm) relative to pulmonary capillaries (14 μm in diameter), which may prevent MSCs from passing through the pulmonary circulation. Researchers found that MSCs injected intravenously into rat stroke models were transiently trapped in the lungs, then were sequestered in the spleen [21]; Minjie Lu *et al. *injected MSCs into the left anterior descending artery of a mini-pig with acute myocardial infarction, and found that the spleen was the main extracardial organ to trap MSCs [22]. One study reported that MSCs were observed in the spleen after intraperitoneal injection [23], but no study has determined whether the trapped MSCs were intact.

ED-1 is a transmembrane protein restricted mainly to monocyte-macrophages. In macrophages, ED-1 is localized mainly in lysosomes and endosomes [24]. We found that MSCs accumulated in the lung were generally of a normal size, and RFP could not co-localize with ED-1. We speculated that these MSCs were intact. While the MSCs accumulated in the spleen were generally fragmented, and most of the RFP could partly or wholly co-localize with ED-1. We speculated that these MSCs were damaged by local macrophages. It was interesting that macrophages in the lung did not alter the survival of MSCs. Several possible reasons may explain this phenomenon. First, MSCs accumulated in the interstitial areas of the lung. Unlike alveolar macrophages which have an active response with enough phagocytes to engulf particles [25], interstitial macrophages are transitional stages between blood macrophages and alveolar macrophages. Interstitial macrophages have a role in limiting inflammation and antigen presentation [25,26]. While MSCs have low immunogenicity and may escape detection by the host immune system upon transplantation, MSCs may also inhibit the activation of macrophages [1,27]. Second, monocyte-derivied macrophages have no proliferative potential in tissues. The macrophage population is maintained by the influx of monocytes from peripheral blood into tissues; the influx rate of monocytes is high in the spleen but low in the lungs [28]. Thus, the function of macrophages in the lung may be relatively stable as they are exposed to fewer influences from peripheral blood. Instead of being phagocytosed by macrophages, MSCs accumulated in the lung might further translocate to the extrapulmonary regional lymph nodes after entering lymphatic capillaries [25]. However, the means by which MSCs injected into the peritoneal cavity accumulated in the spleen shortly after injection remain unclear, as does why our results differed from those of previous studies. Further investigations must be performed to answer these questions.

### MSCs accumulated in the lung after intravenous injection could express TSG-6

We previously examined the cytokine profile in serum-starved MSCs-conditioned medium and found that TSG-6 was released most abundantly. Matrix metalloproteinase (MMP)-8, Fas ligand, vascular endothelial growth factor (VEGF), inter-cellular adhesion molecule (ICAM)-1, some inflammatory and chemotactic factors, were also released by MSCs [11]. TSG-6 has multifunctional anti-inflammatory effects. Transgenic inactivation of the gene increased inflammation, and over-expression of the gene decreased inflammation [12]. We found that TSG-6 secreted by MSCs inhibited inflammation and promoted the repair of mesothelial cells, subsequently reducing peritoneal adhesion [11]. However, we had no direct evidence that MSCs could affect injured peritoneum from a distance. Some studies have reported that cells trapped in the lungs secrete soluble factors into the blood to enhance the repair of other tissues [18].

**Figure 3:**
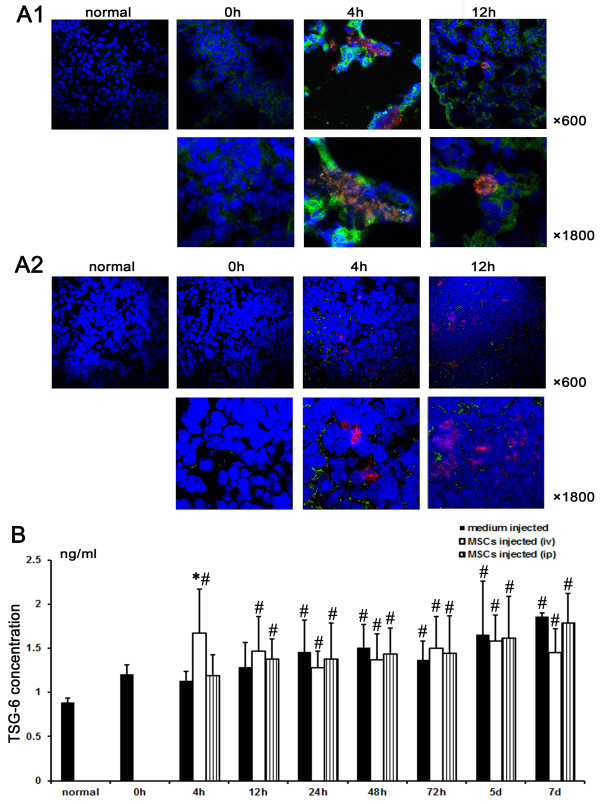
**The expression of TNFα-stimulating gene (TSG)-6 in mesenchymal stem cells (MSCs) injected into rats intravenously**. **A**. The expressions of TSG-6 in MSCs accumulated in the lung and the spleen. **A1**. The majority of MSCs accumulated in the lung expressed TSG-6 within 12 hours, as observed by immunofluorescence microscopy. Red symbolizes red fluorescent protein (RFP); Green symbolizes TSG-6. Magnification: × 600, × 1800. **A2**. The majority of MSCs accumulated in the spleen did not express TSG-6, as observed by immunofluorescence microscopy. Red symbolizes RFP; Green symbolizes TSG-6. Magnification: × 600, × 1800. **B**. The serum TSG-6 concentration was evaluated by enzyme-linked immunosorbent assay (ELISA). Serum TSG-6 level was increased at 4 hours after intravenous injection of MSCs, compared with the medium-injected group. There was no difference between the group injected with MSCs intraperitoneally and the medium-injected group. Three independent samples were placed in three replicate wells. * compared with the medium-injected group, *P *<0.05; # compared with the normal group, *P *<0.05. TNFα, tumor necrosis factor α.

Immunofluorescence microscopy showed that MSCs accumulated in the lung after intravenous injection expressed TSG-6 within 12 hours (Figure 3A1), while MSCs accumulated in the spleen did not express TSG-6 (Figure 3A2). ELISA showed that the concentration of TSG-6 in serum was increased at 4 hours after intravenous injection of MSCs, compared with that in the medium-injected group (Figure 3B) (*P *<0.05, *n *= 3, respectively). The concentration of TSG-6 in serum increased gradually within 7 days after MSCs injection, while the MSCs injection weakened this trend, except for 4 hours after injection (Figure 3B). The changes of serum TSG-6 in the group injected with MSCs intraperitoneally were similar to those in the medium-injected group (Figure 3B).

We previously found that acute inflammation of injured peritoneum peaked at 12 to 24 hours after scraping and the optimal benefit was attained when MSCs were injected at 24 hours after scraping [11]. Therefore, the anti-inflammatory effects of TSG-6 secreted by inflammation-stimulated MSCs were important for their benefits. MSCs accumulated in the lung transiently secreted TSG-6 into the blood, resulting in inhibition of the excessive inflammation of injured peritoneum, which in turn stimulated more weakly the secretion of TSG-6 by MSCs. While the secretion of TSG-6 in the control group increased 48 hours after peritoneal scraping, it might not inhibit the active inflammation efficiently and, thus, failed to reduce peritoneal adhesions.

We found that intraperitoneally injected MSCs were damaged by macrophages in the spleen and did not express or secrete TSG-6 into the blood. So TSG-6 secreted by live MSCs may be a key player that attenuates peritoneal adhesion. These findings may explain the questions we stated at the beginning of this paper.

### MSCs injected intravenously attenuated peritoneal adhesion by secreting TSG-6

**Figure 4:**
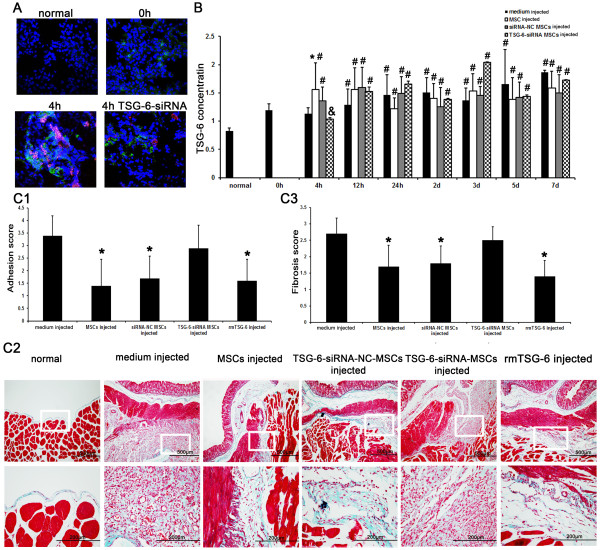
**TNF-stimulating gene (TSG)-6 secreted by mesenchymal stem cells (MSCs) attenuated peritoneal adhesion**. **A**. No TSG-6 expression by TSG-6- small interfering (si) RNA-MSCs in the lung at 4 hours after injection was observed on immunofluorescence microscopy. Red symbolizes red fluorescent protein (RFP); Green symbolizes TSG-6. Magnification: × 600, × 1800. **B**. Serum TSG-6 concentrations as evaluated by enzyme-linked immunosorbent assay (ELISA). No obvious increment of TSG-6 after TSG-6-siRNA-MSCs injection was detected compared with the medium-injected group. The concentration of TSG-6 in the TSG-6-siRNA-MSCs-injected group at 4 hours after injection was lower than that in the MSCs-injected group. Three independent samples were placed in three replicate wells. * compared with the medium-injected group, *P *<0.05; # compared with the normal group, *P *<0.05; & compared with the MSCs-injected group, *P *<0.05. **C**. The effects of TSG-6 on acute peritoneal adhesions. **C1**. The TSG-6-siRNA-MSCs-injected group exhibited no significant reduction in adhesion scores on day 14 after scraping but the recombinant mouse (rm) TSG-6-injected group exhibited an obvious reduction in peritoneal adhesion scores similar to the MSCs-injected group. * compared with the medium-injected group, *P *<0.05 (*n *= 6). **C2**. Histological changes were evaluated using Masson's trichrome staining. The TSG-6-siRNA-MSCs-injected group revealed no reduction in fibrosis in the scraped peritoneum 14 days after scraping. But the rmTSG-6-injected group revealed a distinct reduction in peritoneal fibrosis similar to the MSCs-injected group. Magnification: × 100, × 400. **C3**. The peritoneal fibrosis scores were not reduced by injecting TSG-6-siRNA-MSCs but by injecting rmTSG-6 or MSCs. * compared with the medium-injected group, *P *<0.05 (*n *= 6).

To further evaluate the role of TSG-6 secreted by MSCs in the attenuation of peritoneal adhesion, we knocked down the expression of TSG-6 in MSCs by transient transfection with a TSG-6-siRNA [see Additional file 4, Figure S2]. No TSG-6 expression by TSG-6-siRNA-MSCs in the lung at 4 hours after injection was observed by immunofluorescence microscopy (Figure 4A). ELISA showed no increment of TSG-6 in serum at 4 hours after injection of TSG-6-siRNA-MSCs (Figure 4B). Compared with the medium-injected group, TSG-6-siRNA-MSCs had no significant effect on the size, severity (Figure 4C1) or collagen deposition (Figure 4C2, 4C3) of peritoneal adhesions 14 days after scraping. However, both the size, severity and the collagen depositions of peritoneal adhesions were reduced in rats injected with rmTSG-6 in a manner similar to that in the MSCs-injected group (Figure 4C1, C2, 4C3). Our data demonstrate that TSG-6 secretion by MSCs is necessary for attenuation of peritoneal adhesions.

TSG-6 is a hyaluronan (HA)-binding glycoprotein with multifunctional anti-inflammatory effects. MSCs can block the recruitment of neutrophils by secreting TSG-6 [12] via a CD44/HA/TSG-6 mediated blocking mechanism [29]. Research found that neutrophils of thioglycollate-induced peritonitis were higher in TSG-6-deficient animals than in wild-type animals, but were dramatically suppressed by intravenous injection of rmTSG-6 [30]. MSCs may be activated by an inflammatory microenvironment to secrete TSG-6. In addition, TSG-6/HA/CD44 activates the mitogen-activated protein kinase (MAPK) pathway [31] and enhances the migration and proliferation of injured cells [32]. TSG-6-mediated formation of heavy chain-HA complexes is also involved in remodeling extracellular matrix and regulating cell migration and proliferation [31,33]. We suggest that TSG-6 secreted by MSCs could protect the injured peritoneum from excessive inflammatory response and promote the repair of mesothelial cells, thus reducing the formation of fibrosis [11].

## Conclusions

Our findings suggest that intravenously injected MSCs may accumulate in the lung and attenuate peritoneal adhesion by secreting TSG-6, while intraperitoneally injected MSCs fail to attenuate peritoneal adhesion probably due to phagocytosis by splenic macrophages.

## Abbreviations

ANOVA: one-way analysis of variance; bp: base pair; BSA: bovine serum albumin; ELISA: enzyme-linked immunosorbent assay; FACS: fluorescence-activated cell sorting; HA: hyaluronan acid; ICAM: inter-cellular adhesion molecule; MMP: matrix metalloproteinase; MAPK: mitogen-activated protein kinase; MSCs: mesenchymal stem cells; PD: peritoneal dialysis; RFP: red fluorescent protein; rm: recombinant mouse; RT-PCR: reverse transcriptase-polymerase chain reaction; SD: Sprague-Dawley; siRNA: small interfering RNA; TSG-6: TNFα-stimulating gene-6; VEGF: vascular endothelial growth factor.

## Competing interests

The authors declare that they have no competing interests.

## Authors' contributions

NW conceived and designed the experiments, performed the experiments, analyzed the data, and wrote the paper. YQS performed the experiments, analyzed the data, and wrote the paper. YM conceived and designed the experiments and contributed reagents/materials. LZ conceived and designed the experiments and analyzed the data. QGL conceived and designed the experiments and revised the paper. DGL performed the experiments and analyzed the data. SZS performed the experiments. QH performed the experiments. HLL conceived and designed the experiments, analyzed the data and revised the paper. XMC conceived and designed the experiments, analyzed the data and revised the paper. All authors read and approved the final manuscript.

## Supplementary Material

Additional file 1 **Figure S1**. **Identifications of Sprague-Dawley (SD) rat bone marrow-derived mesenchymal stem cells (MSCs)**. **A**. Representative markers of MSCs. Fluorescence-activated cell sorting (FACS) analysis showed that the positive proportion of cells displaying CD90 was 97.0%, CD54 was 88.8%, CD11a was 10.7% and CD45 was 8.4%. **B**. Multilineage differentiation of MSCs. **B1**. Under osteogenic differentiation conditions, cells displayed extracellular calcium phosphate precipitates as identified by alizarin red staining. Magnification: × 100. **B2**. Under adipogenic differentiation conditions, cells accumulated intracellular lipid droplets as revealed by Oil red staining. Magnification: × 400. Click here for file

Additional file 2 **Video S1**. **Phagocytosis of mesenchymal stem cells (MSCs) 12 hours after intraperitoneal injection by macrophages in the spleen**. MSCs partly co-localized with macrophages in the spleen, as observed by a dynamic three-dimensional image of immunofluorescence microscopy. Red symbolizes RFP; Green symbolizes ED-1 of macrophages. Magnifications: × 1800. Click here for file

Additional file 3 **Video S2**. **Phagocytosis of mesenchymal stem cells (MSCs) 48 hours after intraperitoneal injection by macrophages in the spleen**. MSCs wholly co-localized with macrophages in the spleen 48 hours after intraperitoneal injection, as observed by a dynamic three-dimensional image of immunofluorescence microscopy. Red symbolizes RFP; Green symbolizes ED-1 of macrophages. Magnifications: × 1800. Click here for file

Additional file 4 **Figure S2. Knockdown efficiency of TNFα-stimulating gene (TSG)-6 in mesenchymal stem cells (MSCs)**. **A**. Knockdown efficiency of mRNA in MSCs was approximately 82.9%, as evaluated by reverse-transcriptase polymerase chain reaction (RT-PCR), TSG-6 product length = 134 bp, GAPDH product length = 87 bp. * compared with normal MSCs, *P *<0.05; # compared with 24-hour serum-starved MSCs, *P *<0.05. **B**. Knockdown efficiency of protein in MSCs was approximately 43.5%, as evaluated by Western blot. * compared with normal MSCs, *P *<0.05; # compared with 24-hour serum-starved, *P *<0.05. Click here for file
