# Supplementary figures and images for: Novel mechanism for mesenchymal stem cells in attenuating peritoneal adhesion: accumulating in the lung and secreting tumor necrosis factor α-stimulating gene-6
Source: Stem Cell Res Ther. 2012 Dec 6;3(6):51. doi: 10.1186/scrt142 (PMC3580481; doi:10.1186/scrt142)

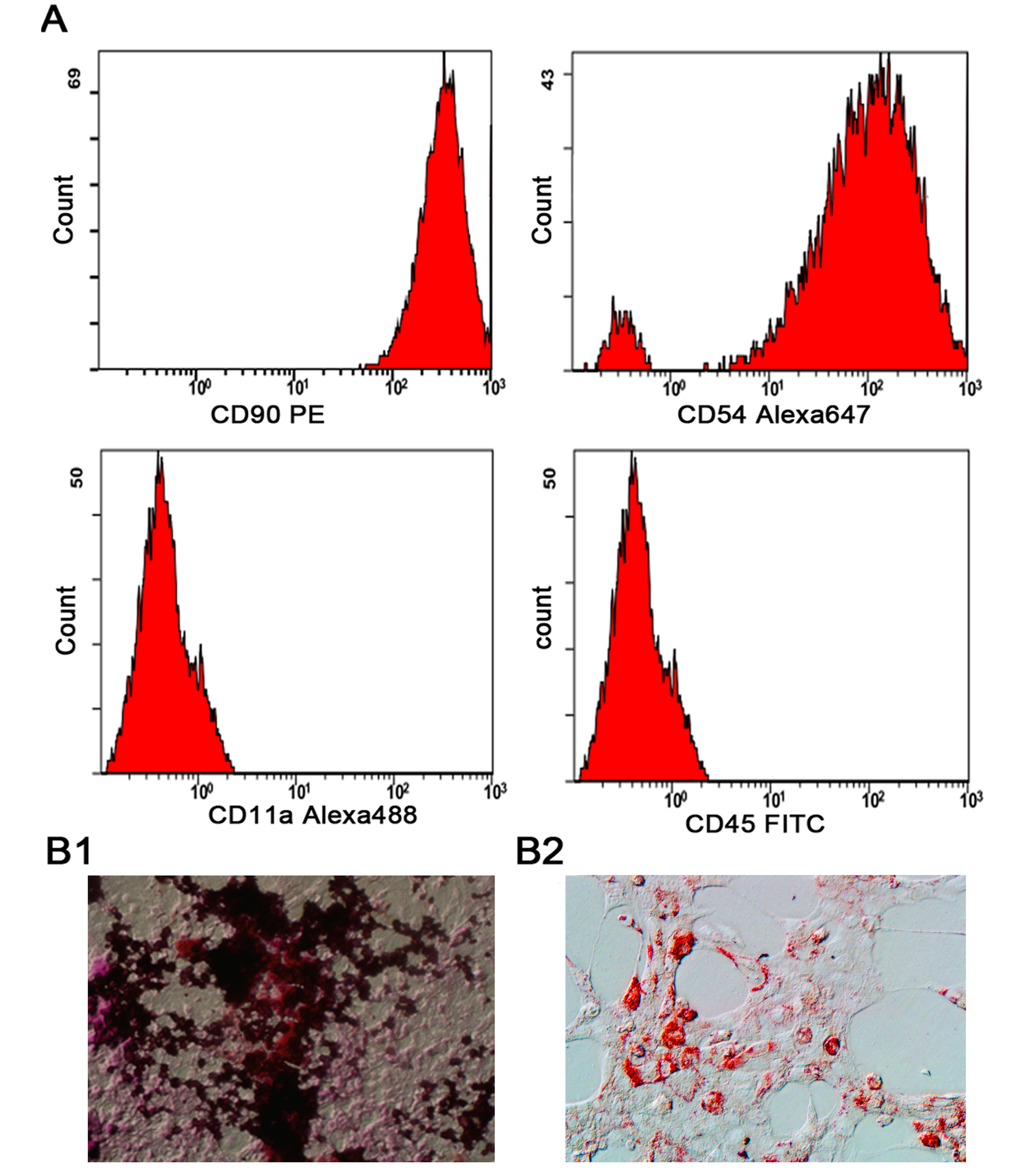

Supplement: Additional file 1 — Figure S1. Identifications of Sprague-Dawley (SD) rat bone marrow-derived mesenchymal stem cells (MSCs). A. Representative markers of MSCs. Fluorescence-activated cell sorting (FACS) analysis showed that the positive proportion of cells displaying CD90 was 97.0%, CD54 was 88.8%, CD11a was 10.7% and CD45 was 8.4%. B. Multilineage differentiation of MSCs. B1. Under osteogenic differentiation conditions, cells displayed extracellular calcium phosphate precipitates as identified by alizarin red staining. Magnification: × 100. B2. Under adipogenic differentiation conditions, cells accumulated intracellular lipid droplets as revealed by Oil red staining. Magnification: × 400. [file scrt142-S1.TIFF]

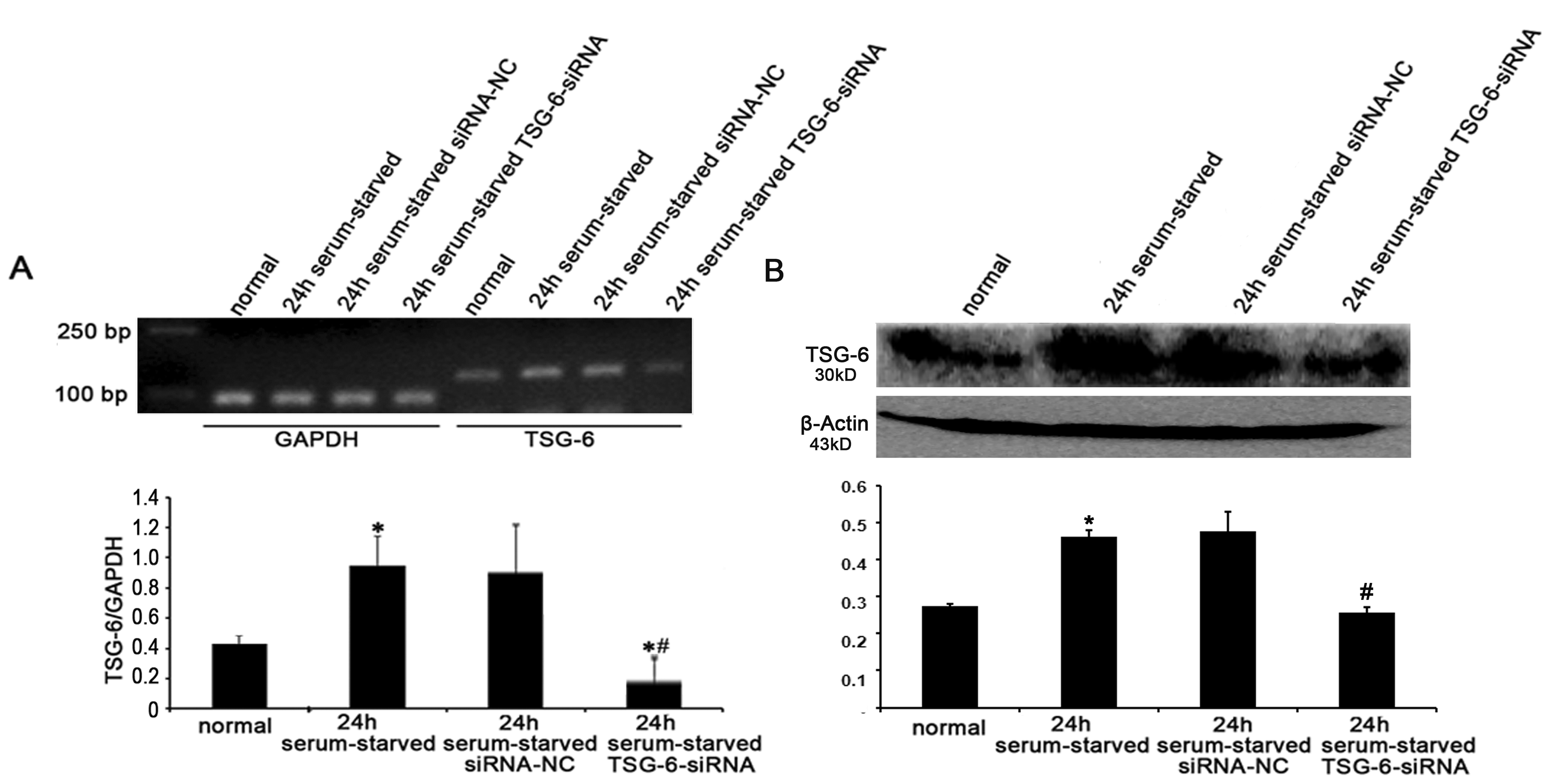

Supplement: Additional file 4 — Figure S2. Knockdown efficiency of TNFα-stimulating gene (TSG)-6 in mesenchymal stem cells (MSCs). A. Knockdown efficiency of mRNA in MSCs was approximately 82.9%, as evaluated by reverse-transcriptase polymerase chain reaction (RT-PCR), TSG-6 product length = 134 bp, GAPDH product length = 87 bp. * compared with normal MSCs, P <0.05; # compared with 24-hour serum-starved MSCs, P <0.05. B. Knockdown efficiency of protein in MSCs was approximately 43.5%, as evaluated by Western blot. * compared with normal MSCs, P <0.05; # compared with 24-hour serum-starved, P <0.05. [file scrt142-S4.TIFF]
